# Planificación para la equidad en la salud en la Región de las Américas: análisis de los planes nacionales de salud^*^

**DOI:** 10.26633/RPSP.2021.106

**Published:** 2021-11-03

**Authors:** Matthew M. Kavanagh, Laura Norato, Eric A. Friedman, Adria N. Armbrister

**Affiliations:** 1 Departamento de Salud Internacional Universidad de Georgetow Washington D.C. Estados Unidos de América Departamento de Salud Internacional, Universidad de Georgetown, Washington, D.C., Estados Unidos de América.; 2 Organización Panamericana de la Salud Washington, D.C. Estados Unidos de América Organización Panamericana de la Salud, Washington, D.C., Estados Unidos de América.

**Keywords:** Equidad en la salud, política pública, política de salud, planes de sistemas de salud, Américas, Health equity, public policy, health policy, health systems plans, Americas, Equidade em saúde, política pública, política de saúde, planos de sistemas de saúde, América

## Abstract

Cada vez más se reconoce que las mejoras en la salud y el bienestar no se han registrado por igual en las poblaciones de la Región de las Américas. En este artículo se analizan 32 políticas, estrategias y planes nacionales del sector de la salud en diez áreas diferentes de la equidad en la salud para comprender, desde una perspectiva, cómo se está abordando el tema de la equidad en la Región. Se encontraron variaciones significativas en la sustancia y estructura de la manera en que los planes de salud manejan el problema. Casi todos los países incluyen explícitamente la equidad en la salud como un objetivo claro y la mayoría de los países abordan los determinantes sociales de la salud. Los procesos participativos documentados seguidos en la formulación de estos planes abarcan desde inexistentes hasta extensos y bien concebidos. Muchos planes incluyen políticas sólidas centradas en la equidad, como las destinadas a mejorar la accesibilidad física de la atención de salud y aumentar el acceso asequible a los medicamentos, pero ningún país incluye todos los aspectos examinados. Los países consideran a las poblaciones marginadas en sus planes, aunque solo una cuarta parte incluye específicamente a los afrodescendientes y más de la mitad no abordan a los pueblos indígenas, incluso algunos con grandes poblaciones indígenas. Cuatro incluyen atención a los migrantes. A pesar de que incluyen objetivos sobre la equidad en la salud y datos sobre las inequidades como parámetros de referencia, menos de la mitad de los países se fijan objetivos con plazos específicos para reducir las desigualdades absolutas o relativas en el ámbito de la salud. Rara vez se encuentran en los planes mecanismos claros de rendición de cuentas, como la educación, la presentación de informes o mecanismos para hacer respetar los derechos. El compromiso casi unánime entre los países de la Región de las Américas con la equidad en la salud ofrece una oportunidad importante. Aprender de los planes más sólidos centrados en la equidad podría proporcionar una hoja de ruta para los esfuerzos tendientes a traducir las metas amplias en objetivos con plazos definidos y, finalmente, aumentar la equidad.

## Abstract (en)

There is growing recognition that health and well-being improvements have not been shared across populations in the Americas. This article analyzes 32 national health sector policies, strategies, and plans across 10 different areas of health equity to understand, from one perspective, how equity is being addressed in the region. It finds significant variation in the substance and structure of how the health plans handle the issue. Nearly all countries explicitly include health equity as a clear goal, and most address the social determinants of health. Participatory processes documented in the development of these plans range from none to extensive and robust. Substantive equity-focused policies, such as those to improve physical accessibility of health care and increase affordable access to medicines, are included in many plans, though no country includes all aspects examined. Countries identify marginalized populations in their plans, though only a quarter specifically identify Afro-descendants and more than half do not address Indigenous people, including countries with large Indigenous populations. Four include attention to migrants. Despite health equity goals and data on baseline inequities, fewer than half of countries include time-bound targets on reducing absolute or relative health inequalities. Clear accountability mechanisms such as education, reporting, or rights-enforcement mechanisms in plans are rare. The nearly unanimous commitment across countries of the Americas to equity in health provides an important opportunity. Learning from the most robust equity-focused plans could provide a road map for efforts to translate broad goals into time-bound targets and eventually to increasing equity.

## RESUMO

É cada vez mais aceito que os avanços em saúde e bem-estar não são partilhados por todas as populações nas Américas. Neste artigo são analisadas 32 políticas, estratégias e planos nacionais de saúde em 10 áreas distintas de equidade em saúde com o objetivo de entender, de uma única perspectiva, como a equidade está sendo contemplada na região. Existem diferenças consideráveis na forma e conteúdo do enfoque dado a esta questão nos planos de saúde. Quase todos os países estabelecem explicitamente a equidade em saúde como uma meta clara e a maioria aborda os determinantes sociais da saúde. O processo participativo documentado na elaboração dos planos também é variável, desde inexistente a amplo e decidido. Muitos planos contêm políticas concretas com foco central em equidade, por exemplo, políticas para melhorar a acessibilidade física à assistência de saúde e o acesso a medicamentos a preços razoáveis, mas nenhum país inclui todos os aspectos aqui examinados. Os países identificam as populações marginalizadas nos seus planos, porém, apenas um quarto distingue especificamente os afrodescendentes e mais da metade não contempla os povos indígenas, mesmo onde as populações indígenas são em grande número. Quatro países consideram a atenção aos migrantes. Embora existam metas de equidade em saúde e dados relativos a iniquidades de base, menos da metade dos países incorpora em seus planos metas com prazos definidos para reduzir as desigualdades absolutas ou relativas em saúde. Instrumentos claros de responsabilidade como educação, prestação de contas ou respeito aos direitos são raramente vistos. O compromisso praticamente unânime dos países das Américas com a equidade em saúde oferece uma oportunidade importante. Os planos mais bem fundados com enfoque em equidade poderiam servir de exemplo para guiar os esforços de converter metas gerais em metas com prazos definidos e, em última instância, aumentar a equidade.

Cada vez se reconoce más que las mejoras en la salud y el bienestar no se han registrado por igual entre las poblaciones. Entre las regiones del mundo, la Región de las Américas tiene una cantidad desproporcionada de contextos muy desiguales, medidos en términos de desigualdad de ingresos (1), acceso a la atención de salud y bienestar (2–7). La evidencia empírica indica que las políticas públicas desempeñan un papel fundamental en la producción o la modificación de los factores que impulsan la inequidad (8). También hay evidencia de que aplicando políticas nacionales específicas se han corregido disparidades en el acceso y el uso de los servicios de salud en la Región (9–11). Sin embargo, no se ha logrado establecer si la buena planificación de las políticas o la programación eficaz son responsables de los avances documentados. En algunas evaluaciones recientes de la equidad en la salud en las políticas se señaló que la planificación adecuada para lograr la equidad y la ejecución de intervenciones a favor de la equidad forman parte de un único indicador asociado con la inequidad en la salud, en tanto que otras sostienen que la formulación de una agenda adecuada, pero sin llevarla a la práctica, puede exacerbar las desigualdades en la salud (12,13). En otras evaluaciones se sostiene que un requisito para lograr la equidad en la salud es la formulación de una política de salud sólida basada en la evidencia (14–16). Recientemente se ha elaborado un sólido marco conceptual de la equidad en la salud en la Región de las Américas y otras regiones, lo que está propiciando un nuevo tipo de planificación y estrategias explícitas para reducir las inequidades en la salud (17,18).

Este artículo responde una pregunta básica para la equidad en materia de salud: ¿están los gobiernos de la Región de las Américas formulando planes sólidos para abordar el tema de la equidad en la salud? Las notables inequidades en la salud podrían deberse a que algunos planes nacionales sólidos no se han puesto en práctica o a que las estrategias para subsanar las inequidades no han tenido éxito. También podría ser que los países no estén teniendo una planificación sólida en torno a la equidad en la salud a nivel nacional. Estos dos contextos sugerirían caminos muy diferentes para los responsables de la toma de decisiones a nivel nacional e internacional que buscan impulsar una salud más equitativa en la Región. Esta cuestión es particularmente aguda en el contexto de la Comisión de la Organización Panamericana de la Salud sobre Equidad y Desigualdades en Salud en las Américas, que instó a los países a “hacer que la equidad en la salud sea un indicador clave del desarrollo de la sociedad y establecer mecanismos de rendición de cuentas”, incluida la planificación de la equidad en la salud (19). ¿Están haciendo esto ya los países? Partiendo de la premisa de que la planificación de políticas es fundamental para reducir las desigualdades en materia de salud, en este artículo se presenta un análisis del estado de integración de la equidad en los planes de salud en la Región de las Américas y de su consonancia con los objetivos clave señalados en el informe de la Comisión. En este análisis se emplea un marco que nos da una perspectiva de la inclusión de la equidad en la salud en 32 planes nacionales junto con una serie de diez categorías que definen acciones prioritarias hacia la equidad en la salud.

Hay variaciones significativas en la sustancia y estructura de la manera en que los planes de salud incorporan la equidad en la salud. Este análisis muestra que algunas áreas y cuestiones de la equidad en la salud se abordan de manera mucho más amplia y sólida, mientras que otras son abordadas por muy pocos países, sin que medie una clara relación con el PIB o cuestiones geográficas. La mayoría de los planes evaluados incluyen el término “equidad en la salud” como parte de la misión o visión del documento y muestran un fuerte enfoque en los determinantes sociales de la salud. Sin embargo, la mayoría no tienen mediciones específicas para evaluar los progresos realizados en la lucha contra las desigualdades ni mecanismos de rendición de cuentas para lograr resultados en materia de equidad en la salud. Las intenciones de abordar la discriminación como un factor impulsor de las desigualdades en la salud son menos frecuentes de lo esperado en los planes.

El objetivo general de este documento es evaluar el grado en que se considera explícitamente abordar el tema de la equidad en la salud en los planes nacionales de salud formulados en los países de la Región de las Américas.

## MATERIALES Y MÉTODOS

Tras examinar la bibliografía y la práctica relativas a la equidad en la salud (16,20), se elaboró un marco para codificar y analizar la inclusión de este concepto en las políticas de salud. Este marco se basa explícitamente en el marco analítico de la Comisión sobre Equidad de la OPS (19,21) y en el trabajo sobre programas de acción para propiciar la equidad en materia de salud, que se han propuesto como un enfoque sistemático para abordar este tema (20). A partir de estos marcos, se seleccionó un conjunto muy amplio pero manejable de 31 indicadores (para un total de 43 preguntas cuando se incluyen las subpreguntas) en diez dominios: 1) misión; 2) determinantes sociales y ambientales de la salud. 3) acciones multisectoriales; 4) procesos participativos; 5) equidad hacia la salud universal; 6) inclusión de grupos poblacionales tradicionalmente excluidos; 7) datos desglosados y metas; 8) seguimiento; 9) rendición de cuentas, y 10) capacidad para responder a las desigualdades en la salud. En el cuadro 1 más adelante se proporcionan todas las preguntas y todos los indicadores. Los dominios siguen la conceptualización descrita anteriormente que centra el proceso y los resultados, y abarcan el ciclo de políticas, desde la formulación del plan hasta dominios clave de su contenido, seguimiento y evaluación, rendición de cuentas y más investigaciones para mejorar las políticas. Con base en Creswell y Poth (22), se verificó el marco por medio de algunos enfoques de refuerzo como la revisión por pares y opiniones de los informantes. La revisión por pares estuvo a cargo de un pequeño grupo asesor de expertos de la Organización Panamericana de la Salud (OPS) y de la Universidad Johns Hopkins, y el segundo enfoque de refuerzo tuvo lugar mediante una serie de publicaciones y un ciberseminario con varios cientos de participantes que tuvo lugar en enero de 2019.

Con el apoyo de las representaciones y el personal de la OPS, se recogieron las políticas, las estrategias y los planes nacionales por escrito más recientes del sector de la salud de los países de la Región de las Américas. La Organización Mundial de la Salud (OMS) ha instado a todos los países a que crearan políticas, estrategias y planes nacionales coherentes, en forma de un documento específico de política nacional de salud, bajo la premisa de que la formulación de estrategias, que implica diseñar planes y políticas para lograr un objetivo particular relacionado con la salud de una nación, es absolutamente crucial en el siglo XXI (8). Esos planes, como describe la OMS, deben ser intersectoriales y abordar tanto la salud como la equidad en la salud dentro del proceso nacional general de planificación sanitaria. La mayoría de los países de la Región tienen un documento oficial de políticas, estrategias y planes nacionales para el sector de la salud elaborado por el gobierno y las representaciones de la OPS pudieron compartirlos a petición o verificar si un país no había elaborado ese documento. Se reunieron políticas, estrategias y planes nacionales para el sector de la salud de 32 países y territorios de la OPS (véase el Anexo 1 en los materiales complementarios). El texto de esos documentos constituyó la base del análisis de este artículo. Canadá, Estados Unidos de América y Cuba no fueron incluidos en este análisis porque en el momento del examen, en diciembre del 2019, estos países no tenían un único plan nacional de salud que pudiera codificarse, como era el caso en otros países de la Región.^1^ Algunas alternativas como la legislación nacional de salud no pueden compararse con los planes nacionales (por ejemplo, porque se centran en temas más específicos como los seguros de salud) y, en consecuencia, no se pueden comparar de manera significativa con otros países.

**CUADRO 1.** Equidad en la salud en los planes nacionales de salud

**Sección**	**Indicador y pregunta**		**Puntuación de la pregunta**	**Puntuación de la sección**
**1. Misión**	A. ¿Se incluye la equidad en la salud como parte de la misión o visión del plan de salud? (Sí/No)		1	1
**2. Determinantes sociales y ambientales de la salud**		a. ¿Incorpora el plan de salud medidas para mejorar los determinantes subyacentes de la salud, como un mayor acceso a alimentos nutritivos, agua potable, saneamiento mejorado o entornos más saludables?	1	3
		b. ¿Se incluye en el plan modelos de financiamiento para incentivar la acción del sector de la salud sobre los determinantes sociales de la salud?	1	
		c. ¿Se incluye en el plan acciones que está tomando el sector de la salud para responder al cambio climático?	1	
**3. Acciones multisectoriales**	A. ¿Se incluye en el plan de salud alguna medida para abordar la equidad en la salud en el sector privado?		1	1
**4. Procesos participativos**	A. ¿Se utilizaron procesos o mecanismos participativos para formular el plan nacional de salud?	a. ¿En el plan de salud se hace referencia o se describe un proceso en su formulación que haya incluido participación pública, de la sociedad civil o ambas?	1	5
		b. Si la respuesta a la pregunta 3A es afirmativa, ¿se refiere el plan a la extensión o inclusión específica de grupos poblacionales marginados?	1	
		c. Si la respuesta a la pregunta 3A es afirmativa, ¿se refirió el proceso a la participación de sectores no sanitarios en la formulación del plan?	1	
	B. ¿Se incluye en el plan nacional de salud algún proceso o mecanismo participativo para formular e implementar políticas y programas de salud?	a. ¿Se refiere el plan de salud a la importancia de la participación pública en la formulación y la aplicación, y menciona mecanismos específicos para la participación pública (o de la sociedad civil)?	1	
		b. Si la respuesta a la pregunta 3Bi es afirmativa, ¿se incluye en el plan de salud alguna acción para apoyar el funcionamiento de estos mecanismos (por ejemplo, financiamiento, capacitación, extensión a grupos poblacionales marginados)?	1	
**5. Equidad hacia la cobertura universal de salud**	A. ¿Se incluye en el plan nacional de salud acciones para lograr la equidad dentro del sector de la salud?	a. No discriminación 1. ¿Incluye o se refiere el plan de salud a una estrategia para abordar la discriminación en el sector de la salud?	1	7
		b. Accesibilidad física ¿Se incluye en el plan de salud al menos una acción (que no esté relacionada con el personal de salud) para aumentar la accesibilidad a servicios de atención primaria de salud de calidad en zonas geográficas o comunidades remotas, rurales o de otra manera desatendidas (por ejemplo, construcción de centros de salud en esas zonas, dispensarios móviles, telemedicina)? ¿Se incluye en el plan de salud al menos una acción para garantizar la accesibilidad a los centros de salud para las personas con discapacidad?	0,5 (ii.1) 0,5 (ii.2)	
		c. Personal de salud ¿Se incluye en el plan de salud acciones para incrementar el número de trabajadores de salud en las comunidades desatendidas? ¿Se incluye en el plan de salud alguna acción con respecto a la contratación de personas de comunidades subrepresentadas en el personal de salud, inclusive en puestos directivos u otros cargos de autoridad?	0,5 (iii.1) 0,5 (iii.2)	
		d. Financiamiento para la salud ¿Se incluye en el plan de salud intervenciones para aumentar la asequibilidad de los servicios de salud para los grupos poblacionales desfavorecidos (por ejemplo, desvincular de los costos el uso de los servicios de salud para estos grupos, otorgar subsidios)? ¿Se incluye en el plan de salud estrategias para propiciar la distribución equitativa del financiamiento para la salud (por ejemplo, más recursos a las comunidades con peores resultados de salud, grupos poblacionales más desfavorecidos)?	0,5 (iv.1) 0,5 (iv.2)	
		e. Información sanitaria ¿Se incluye en el plan de salud alguna acción para mejorar la alfabetización sanitaria de las poblaciones marginadas? ¿Se considera en el plan de salud los obstáculos lingüísticos a los servicios de salud (por ejemplo, servicios de interpretación, contratación de personal sanitario de minorías lingüísticas)?	0,5 (v.1) 0,5 (v.2)	
		f. Medicamentos y tecnologías médicas (desabastecimiento o cadena de suministro en zonas desatendidas, asequibilidad) ¿Se incluye en el plan de salud intervenciones para aumentar el acceso de los grupos poblacionales marginados a los medicamentos (por ejemplo, mayor asequibilidad, mejora de las cadenas de suministro para reducir el desabastecimiento en zonas remotas)?		
	B. ¿Se incluye en el plan de salud un objetivo de cobertura universal de salud?		1	
**6. Inclusión de poblaciones marginadas**	A. ¿Se consideran en el plan nacional de salud grupos poblacionales marginados específicos?	a. ¿Se identifican en el plan de salud grupos poblacionales marginados específicos que enfrentan obstáculos adicionales a la igualdad en la salud? (Sí/No) ¿Se incluye a los afrodescendientes en los grupos considerados?	1 (Nota: Se acredita un punto por identificar la población en su conjunto; se registra, pero no se otorga puntuación para los casos que se incluyen los grupos ya que hay países que tienen distintas combinaciones de grupos a identificar según cada caso)	3
		a. ¿Se identifican en el plan de salud grupos poblacionales marginados específicos que enfrentan obstáculos adicionales a la igualdad en la salud? (Sí/No) 2. ¿Se incluye a pueblos indígenas en los grupos considerados? a. ¿Se identifican en el plan de salud grupos poblacionales marginados específicos que enfrentanobstáculos adicionales a la igualdad en la salud? (Sí/No) 3. ¿Se incluye a los pueblos romaníes en los grupos considerados? a. ¿Se identifican en el plan de salud grupos poblacionales marginados específicos que enfrentan obstáculos adicionales a la igualdad en la salud? (Sí/No) 4. ¿Se incluye a personas con discapacidad en los grupos considerados? a. ¿Se identifican en el plan de salud grupos poblacionales marginados específicos que enfrentanobstáculos adicionales a la igualdad en la salud? (Sí/No) 5. ¿Se incluye a miembros de la comunidad LGBTI en los grupos considerados? a. ¿Se identifican en el plan de salud grupos poblacionales marginados específicos que enfrentanobstáculos adicionales a la igualdad en la salud? (Sí/No) 6. ¿Se incluye a los migrantes en los grupos considerados? a. ¿Se identifican en el plan de salud grupos poblacionales marginados específicos que enfrentan obstáculos adicionales a la igualdad en la salud? (Sí/No) 7. ¿Se incluye a personas que viven en situación de pobreza en los grupos considerados? a. ¿Se identifican en el plan de salud grupos poblacionales marginados específicos que enfrentanobstáculos adicionales a la igualdad en la salud? (Sí/No) 8. ¿Hay otros grupos que vivan en situación de vulnerabilidad según el contexto nacional entre los grupos que se identificaron?		
		b. ¿Incluye el plan de salud medidas específicas para reducir los obstáculos a la buena salud de los grupos marginados consideradas?	1	
		c. ¿Se refiere el plan de salud a algunas acciones para asegurar que se diferencien los programas y servicios a fin de satisfacer las necesidades distintas de las mujeres, las niñas, los hombres y los niños?	1	
**7. Datos desglosados y objetivos**	A. ¿Se incluye en el plan de salud la recopilación de datos desglosados y se utilizan estos datos para establecer objetivos?	a. ¿Se incluye en el plan datos de referencia sobre inequidades en la salud en múltiples dimensiones (por ejemplo, ingresos, género, edad, raza, etnia, condición de indígena, condición migratoria, discapacidad, ubicación geográfica)?	1	3
		b. Si se presentan datos desglosados, ¿se incluyen en el plan de salud los datos desglosados según las dimensiones señaladas en la meta 17.18 de los Objetivos de Desarrollo Sostenible (ingresos, género [sexo], edad, raza, etnia, condición migratoria, discapacidad, ubicación geográfica y otras características relevantes en contextos nacionales)?	1	
		c. ¿Se incluye en el plan de salud objetivos con plazos específicos para reducir las desigualdades absolutas o relativas en materia de salud en el acceso a los servicios de salud (cobertura) o en los resultados de salud?	1	
**8. Seguimiento**	A. ¿Se incluye en el plan de salud procesos para dar seguimiento a los progresos realizados en su implementación?	a. ¿Se incluye en el plan de salud algún proceso para seguimiento y evaluación periódicos de sus objetivos y metas?	1	3
		b. ¿Es el plan de salud fácilmente accesible al público? ¿Está el plan de salud disponible en línea? ¿Se incluye en el plan de salud alguna estrategia para comunicar el contenido al público, incluidos los miembros de las comunidades marginadas?	0,5 (ii.1) 0,5 (ii.2)	
		c. ¿Se incluye en el plan de salud la participación del público en el seguimiento y evaluación de su implementación?	1	
**9. Rendición de cuentas**	A ¿Se incluye en el plan de salud mecanismos para reparar violaciones del derecho de las personas a la salud?	a. ¿Se incluye en el plan de salud mecanismos para educar a las personas sobre su derecho a la salud?	1	4
		b. ¿Se incluye en el plan de salud mecanismos para notificar violaciones del derecho a la salud?	1	
		c. ¿Se incluye en el plan de salud mecanismos para hacer respetar el derecho de las personas a la salud?	1	
		d. ¿Se incluye en el plan mecanismos para investigar y reducir el fraude y la corrupción?	1	
**10. Capacidad para responder a las desigualdades en la salud**	A. ¿Se incluye en el plan de salud algunas acciones de investigación para comprender mejor y abordar las desigualdades en la salud?		1	1
**Total**				31

***Fuente:*** preparado por los autores sobre la base de los resultados del estudio.

Si bien los planes que se analizaron no representan una imagen completa de las políticas de salud de los países, constituyen una perspectiva de los objetivos, intenciones y enfoques que los gobiernos nacionales están adoptando dentro del sistema de salud en un momento dado. Por consiguiente, en este artículo se hace una incursión inicial en la codificación y el análisis de las políticas de equidad en la salud y debe verse desde esa óptica. Tras una aplicación inicial del marco se obtuvo más información y se le hicieron ligeros cambios.

Cada uno de los planes recopilados fue codificado en cada uno de los indicadores utilizando una codificación binaria de 0 o 1 para señalar si cada factor estaba o no presente en el plan. A las subpreguntas se les otorgaron puntuaciones fraccionarias para que el total de cada pregunta fuera igual a 1 (véase el cuadro 1). A las subpreguntas relacionadas con los grupos poblacionales considerados en los planes de salud no se les asignó una puntuación, porque la decisión de incluir o no un grupo poblacional determinado depende del contexto, aunque de todas maneras se informan más adelante.

## RESULTADOS

### Hallazgos generales: Variación entre los países

El grado en que los países habían incorporado la equidad en sus planes nacionales de salud variaba considerablemente, como se muestra en el cuadro 2, que ofrece una tabulación de la parte de las preguntas que los países respondieron afirmativamente, lo que indica la inclusión de políticas para promover la equidad en la salud. En general, El Salvador, Colombia, Chile, Honduras y Uruguay fueron los que incluyeron más elementos del marco, pero ningún país incluyó todas las partes. La puntuación más alta fue 22 de 31 indicadores codificados “sí”. Sin embargo, aparte de las preguntas sobre la contratación de personas subrepresentadas en la fuerza laboral y los modelos de financiamiento para los determinantes sociales de la salud, al menos unos pocos países respondieron “sí” a cada pregunta.

La forma en que los países incorporaron la equidad varía considerablemente. Por ejemplo, Chile, Colombia y Guyana incluyeron muchos de los indicadores de participación en sus planes, mientras que países como la República Dominicana, México, y San Vicente y las Granadinas lo hicieron en menor medida. Brasil, El Salvador y Honduras se centraron menos en la participación en sus planes, pero incluyeron muchos de los elementos de la salud universal y la atención de salud para todos. A diferencia de la mayoría de los países, Belice, Chile, San Kitts y Nevis y Suriname incluyeron los tres elementos codificados en el desglose de datos y objetivos.

### Misión, visión y determinantes sociales de la salud

Casi todos los países, 30 de los 32, incluyeron la equidad en la salud como parte de la misión o visión de su plan de salud (o en otras secciones del documento). La Estrategia Nacional de Salud para el Cumplimiento de los Objetivos Sanitarios 2011-2020 de Chile es ejemplo de ello, dado que su quinto objetivo estratégico es reducir las desigualdades en la salud de la población mitigando los efectos producidos por los determinantes sociales y económicos de la salud.

**FIGURA 1.**
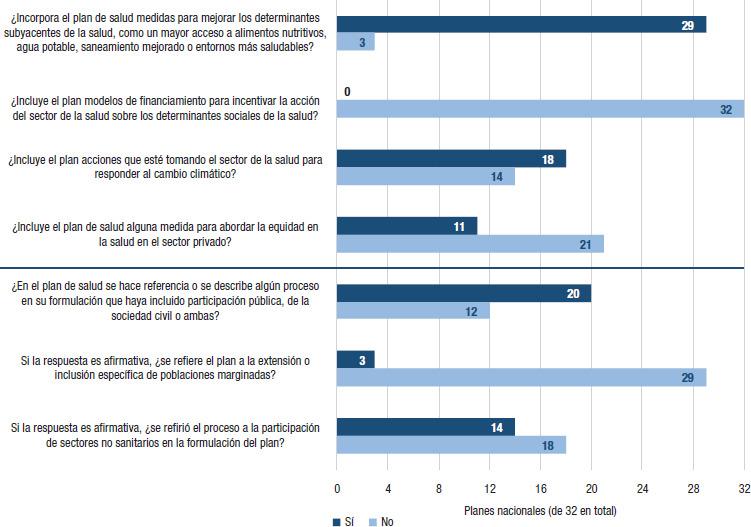
Inclusión de los determinantes sociales y ambientales de la salud y participación en 32 planes nacionales de salud

Asimismo, en 30 de los 32 planes de salud de los países se abordan los determinantes subyacentes de la salud, como un mayor acceso a alimentos nutritivos, agua potable, saneamiento mejorado o entornos más saludables (figura 1). El plan nacional de salud de Barbados ejemplifica una respuesta afirmativa a esta pregunta. Su plan incluye acciones relacionadas con la alimentación y la nutrición, y el acceso a agua y saneamiento. También incluye metas específicas para este objetivo, con el compromiso de reducir en 50% la dependencia de las importaciones de alimentos y desarrollar un programa nacional de seguridad alimentaria para el 2010.

A diferencia de los frecuentes resultados positivos con respecto a las medidas para mejorar los determinantes subyacentes de la salud, ningún país tenía modelos de financiamiento que incentivaran la consideración de los determinantes sociales de la salud. Sin embargo, en el plan estratégico del sector de la salud de Belice para el período 2014-2024 se aborda el financiamiento de la seguridad vial y se menciona un mecanismo financiero para un proyecto de seguridad vial mediante un préstamo del Banco Interamericano de Desarrollo.

En varias otras secciones en el dominio de los determinantes sociales de la salud, el panorama es más complejo. Poco más de la mitad (18 de 32) de los planes de los países analizados incluyen medidas para responder al cambio climático. Mientras tanto, pese a que el sector privado desempeña un papel cada vez mayor en muchos países, pocos fueron los que abordaron la equidad en la salud en el sector privado en sus planes de salud. Solo 11 de los 32 planes de salud lo hicieron.

### Participación en el diseño e implementación de los planes

En más de la mitad de los planes de los países analizados (20 de 32) se describe un proceso de formulación del plan que incluyó la participación pública, de la sociedad civil o de ambas (figura 1). Por ejemplo, para elaborar el Plan Decenal de Salud Pública 2012-2021 de Colombia se recurrió a una consulta pública durante el proceso de diseño. Sin embargo, pocos planes de salud se refieren actividades de extensión para llegar a grupos poblacionales marginados específicos (u otros).

**CUADRO 2.** Inclusión de la equidad en los planes nacionales de salud según su puntuación en los 31 indicadores sobre los diez dominios

	**1. Misión**	**2. Determinantes sociales y ambientales de la salud**	**3. Acciones multisectoriales**	**4. Procesos participativos**	**5. Equidad hacia la salud universal**	**6. Inclusión de grupos poblacionales tradicionalmente excluidos**	**7. Datos desglosados y metas**	**8. Seguimiento**	**9. Rendición de cuentas**	**10. Capacidad para responder a las inequidades en la salud**		***Puntuación total / Clasificación***	
***Valor máximo / Puntuación***	1	3	1	5	7	3	3	3	4	1		31	100%
**Antigua y Barbuda**	1	2	1	2	2	2	0	1,5	0	0		11,5	37,1
**Argentina**	1	1	0	1	1	0	0	1	0	0		5	16,1
**Bahamas**	1	2	0	2	3	1	0	2,5	1	1		13,5	43,5
**Barbados**	1	2	0	2	1	1	0	0,5	0	0		7,5	24,2
**Belice**	1	0	1	3	2	1	3	2,5	0	1		14,5	46,8
**Bolivia (Estado Plurinacional de)**	1	2	0	3	4,5	2	2	1,5	1	0		17	54,8
**Brasil**	1	1	0	2	4,5	2	0	2,5	2	0		15	48,4
**Chile**	1	2	0	4	4	2	3	3	0	0	19		61,3
**Colombia**	1	2	0	5	4	3	2	2,5	0	0		19,5	62,9
**Costa Rica**	1	2	0	2	2	1	1	2	1	0		12	38,7
**Dominica**	1	1	0	3	2,5	2	2	2,5	0	0		14	45,2
**República Dominicana**	1	1	0	0	1,5	2	2	2	0	0		9,5	30,6
**Ecuador**	1	1	0	3	2,5	2	2	2	0	0		13,5	43,5
**El Salvador**	1	2	1	3	5,5	3	0	2,5	3	1		22	71,0
**Granada**	1	2	0	3	3	3	1	0,5	0	0		13,5	43,5
**Guatemala**	1	1	0	1	1,5	2	0	1,5	0	0		8	25,8
**Guyana**	1	1	1	4	5	2	0	3	0	0		17	54,8
**Haiti**	1	1	1	2	3	3	1	1,5	1	1		15,5	50,0
**Honduras**	1	2	1	3	4,5	3	2	1,5	1	0		19	61,3
**Jamaica**	1	2	1	3	3,5	2	2	1,5	0	0		16	51,6
**México**	1	1	0	0	2,5	2	1	1,5	0	0		9	29,0
**Nicaragua**	1	1	0	1	1,5	2	0	1,5	1	0		9	29,0
**Panamá**	1	2	0	1	2,5	2	2	3	1	1		15,5	50,0
**Paraguay**	1	2	0	3	2,5	1	0	0,5	0	0		10	32,3
**Perú**	1	2	0	2	4,5	2	2	2,5	0	0		16	51,6
**San Kitts y Nevis**	1	1	1	3	3,5	2	3	1	0	1		16,5	53,2
**Santa Lucía**	1	2	0	2	0	0	0	1,5	1	0		7,5	24,2
**San Vicente y las Granadinas**	0	2	0	0	0,5	0	1	1	0	0		4,5	14,5
**Suriname**	1	2	1	2	2,5	1	3	1,5	1	1		16	51,6
**Trinidad y Tabago**	1	1	1	1	1	0	1	2	0	0		8	25,8
**Uruguay**	1	1	1	3	5,5	3	2	2	1	0		19,5	62,9
**Venezuela (República Bolivariana de)**	0	0	0	1	3	1	1	0	0	0		6	19,4
									**Puntuación total**				31
									**Puntuación promedio**				13,13
									**Puntuación mínima**				4,5
									**Puntuación máxima**				22

***Fuente:*** preparado por los autores sobre la base de los resultados del estudio.

Resulta alentador que la mayoría de los planes nacionales de salud reconocen la necesidad de la participación pública y se refieren a mecanismos específicos para que intervenga el público (o la sociedad civil) en la formulación y aplicación de políticas y programas (28 de 32 países). La política nacional de salud de Guyana es ejemplar. Incluye principios rectores adicionales dedicados a la participación social activa e incorpora un comité nacional de políticas de salud como mecanismo para incluir a la sociedad civil y a las organizaciones del sector privado en el fortalecimiento del marco legislativo, institucional y de política del sistema de salud.

***Fuente:*** preparada por los autores sobre la base de los resultados del estudio.

Sin embargo, se encontraron pocas referencias de que estos mecanismos participativos fuesen esfuerzos estructurados y financiados. Brasil es uno de los cinco países (de 32) que incluyen acciones para apoyar el funcionamiento de estos mecanismos, por ejemplo, al apoyar el establecimiento de estructuras descentralizadas de defensor del pueblo, aplicar políticas para fomentar la evaluación de los servicios por parte de los usuarios, y difundir información sobre el derecho a la salud y el ejercicio de ese derecho.

### Equidad hacia la salud universal y la atención de salud para todos

En la mayoría de los países, los planes mostraron una gran diversidad en la forma en que abordaron la salud y la atención de salud en general, inclusive en cuanto a las medidas cruciales para la equidad en la salud. La mayoría de los planes (23) incluyen un objetivo para proporcionar cobertura universal de salud. Sin embargo, los pasos específicos hacia la equidad para garantizar la atención de salud para todos son menos frecuentes. Las áreas más comunes consideradas por los planes en este tema fueron los medicamentos —poco menos de la mitad de los planes incluyen intervenciones para aumentar el acceso de los grupos poblacionales marginados a los medicamentos (por ejemplo, propiciar la asequibilidad, reducir el desabastecimiento en zonas remotas)— y la accesibilidad física, con un poco más de la mitad de los planes que incluyen al menos una acción para aumentar la accesibilidad a servicios de atención primaria de salud de calidad en zonas geográficas o comunidades remotas, rurales o de otra manera desatendidas.

Diez de los 32 países incluyeron acciones para incrementar el número de trabajadores de la salud en comunidades desatendidas, aunque solo el plan de Jamaica contenía medidas para reclutar a personas de comunidades subrepresentadas en el personal de salud. Un número similar incluye intervenciones para aumentar la asequibilidad de los servicios de salud para las poblaciones desfavorecidas (14 países) así como intervenciones para facilitar la distribución equitativa del financiamiento para la salud (13 países).

### Discriminación e identificación de los grupos poblacionales en situación de vulnerabilidad

Once de 32 planes de salud de los países incorporan o se refieren a una estrategia para abordar el tema de la discriminación en el sector de la salud. El plan de salud de Costa Rica ofrece un buen ejemplo, con dos estrategias que se aplicarán en todo el sector de la salud para abordar la desigualdad de género y la violencia contra las personas LGBTI.

**FIGURA 2.**
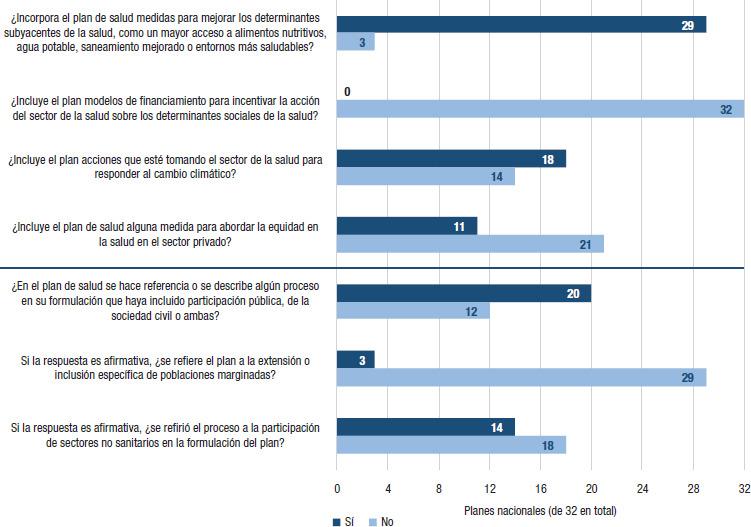
Inclusión de grupos poblacionales en situación de vulnerabilidad en 32 planes nacionales de salud

LGBTI, lesbianas, gais, bisexuales, transexuales e intersexuales.

***Fuente:*** preparada por los autores sobre la base de los resultados del estudio.

A pesar del pequeño número de planes de salud que incluyen estrategias para eliminar la discriminación, en la mayoría de los planes examinados se hace referencia a múltiples grupos poblacionales que enfrentan obstáculos para la igualdad en la salud. En la figura 2 se muestra el número de países que mencionan cada una de las ocho categorías de población en su plan nacional. Las personas que viven en la pobreza y las personas con discapacidad son los dos grupos socialmente excluidos que más a menudo se mencionan en los planes de salud. Los pueblos romaníes y los migrantes son los menos mencionados. Reconociendo la posibilidad de que algunos países no alberguen a ciertas poblaciones –por ejemplo, muchos países simplemente podrían no tener una población romaní– se evaluó la inclusión de estas poblaciones, pero no se la tuvo en cuenta en la puntuación. El análisis para establecer si se trata de una exclusión justificable o si es una omisión que debería rectificarse requiere una evaluación del contexto del país que está fuera del alcance de este estudio.

### Datos, seguimiento y rendición de cuentas

Más de la mitad de los planes analizados (19 de 32) incluyen datos de referencia sobre las inequidades en la salud en múltiples dimensiones (por ejemplo, ingresos, género, edad, raza, etnia, condición migratoria, discapacidad, ubicación geográfica). Cuarenta y un por ciento de los planes (13 de 32) tienen metas con plazos específicos para reducir las desigualdades absolutas o relativas en la salud en cuanto al acceso a servicios de salud o a los resultados de salud. Panamá es un buen ejemplo, su Política Nacional de Salud y Lineamientos Estratégicos 2016-2025 analiza la situación de la salud del país con datos desglosados sobre varios temas relacionados con la salud.

El número de estrategias nacionales de desarrollo que incluyen indicadores o metas de equidad en la salud con plazos específicos es menor. Casi la mitad de los países (15 de 32) incluyen indicadores específicos y metas con plazos determinados (en algunos casos, todavía están en elaboración) para la salud en general, aunque solo aproximadamente la mitad de estos países (ocho) incluyen uno o varios indicadores o metas relacionadas con la equidad. De los ocho países que incluyen metas de equidad en la salud en sus estrategias nacionales de desarrollo, seis también tienen esas metas en sus planes nacionales de salud (de los 13 planes nacionales de salud que contienen esas metas).

Ochenta y cuatro por ciento de los planes (27 de 32) incorporan un proceso de seguimiento y evaluación periódicos de sus objetivos y metas. Sin embargo, solo el 31% (10 de 32 países) incluye la participación del público en el seguimiento y evaluación de la implementación del plan de salud. El grado de especificidad de los procesos de seguimiento y evaluación varía. Por ejemplo, Honduras incluye una descripción general del proceso de seguimiento, y el plan de salud de Suriname describe al seguimiento como un objetivo del propio plan y especifica metas para cumplirlo, junto con la creación de dos comités de seguimiento e implementación.

Muy pocos planes de salud de los países abordaron mecanismos de rendición de cuentas vinculados al derecho a la salud. Solo los planes de salud de dos países describen mecanismos para denunciar violaciones del derecho a la salud, y solo tres mencionan mecanismos para investigar y reducir el fraude y la corrupción.

## DISCUSIÓN

Dado su propósito de determinar si los países de la Región de las Américas están planificando abordar la equidad en la salud, este estudio arroja resultados mixtos, con razones significativas para creer que se podría fortalecer la planificación. Las recomendaciones de la Comisión sobre Equidad de la OPS se apoyan en cambios a nivel del gobierno que comienzan con un llamado a formular planes estratégicos para mejorar la equidad en la salud (19). Tras examinar los planes nacionales del sector de la salud se observa que efectivamente que esto es una brecha en todos los países de la Región, aun cuando muchos ya hayan tomado piezas clave de esta labor y podrían intercambiar sus experiencias con otros países.

Las recomendaciones de la Comisión sobre Equidad aún no están plasmadas en los planes actuales del sector de la salud en la Región de las Américas. Sin embargo, hay razones para mostrarse optimistas, ya que los países están prestando atención a la equidad en la salud en sus políticas, estrategias y planes nacionales por escrito para el sector de la salud. Casi todos los países incluyen explícitamente la equidad en la salud como un claro objetivo de esos planes.

Según lo recomendado por la OPS, la gran mayoría incluye una atención específica a los determinantes sociales de la salud en sus planes. Dado que en muchos países de la Región el sector privado desempeña un papel cada vez más importante, lo que tiene implicaciones significativas para la equidad, llama la atención que en pocos planes se haga referencia a ese sector.

La atención a la participación varía mucho entre los países: un país incluye todos los indicadores y unos pocos países incluyen muchos de ellos, pero otros no incluyen ninguno.

Ningún país incluye todas las medidas de verdadera equidad en los sistemas de salud, pero algunos incluyen varias de estas medidas, como mejorar la accesibilidad física y financiera, y ampliar el acceso a los medicamentos para los grupos poblacionales socialmente excluidos. Fueron menos los países que abordaron otros aspectos, como la discriminación, un mayor acceso a los trabajadores de salud en las zonas desatendidas y la eliminación de obstáculos lingüísticos. En general, los países incluyen la atención a los grupos poblacionales socialmente excluidos en sus planes de salud, aunque con algunas limitaciones que cabe destacar: solo una cuarta parte de los planes mencionan a los afrodescendientes, menos de la mitad incluyen a los pueblos indígenas, y algunos países con grandes poblaciones indígenas no los toman en cuenta. Solo cuatro países de la Región incluyen la atención de los migrantes.

Una minoría de países, solo el 41%, incluye objetivos con plazos específicos para reducir las desigualdades absolutas o relativas en materia de salud. Curiosamente, el establecimiento de objetivos con plazos determinados se corresponde con bastante frecuencia con los países con sólidos planes de equidad en todos los ámbitos. Sin embargo, hay excepciones, entre ellas México, que establece metas claras para la equidad en la salud, aunque el país solo incluye 10 de 31 indicadores en el plan de salud nacional. Al igual que en el caso de El Salvador, algunos países con los planes más robustos aún no han fijado objetivos de equidad con plazos específicos.

En general, muy pocos países incluyeron mecanismos claros de rendición de cuentas que podríamos esperar ver en los planes que abordan la equidad en lo que respecta a la salud, con solo unas pocas referencias a la educación, la presentación de informes o los mecanismos de observancia en este ámbito.

Cabe destacar que de los 32 países cuyos planes de salud analizamos, la puntuación media en este marco es de solo 13 indicadores de 31 que fueron incluidos, y que ningún país ha incluido más del 70%. En toda la Región hay mucho por hacer en cuanto a la planificación. También cabe destacar el hecho de que algunos países con mejores resultados en el ámbito de la salud, como Argentina, presten relativamente poca atención a la equidad en la salud en sus planes nacionales de salud, mientras que otros, como Haiti, con la esperanza de vida más baja de la Región, le presten mucha más atención. Por supuesto, no existe una simple línea causal entre el contenido de los planes por escrito, que es el principal enfoque de este estudio, y los resultados de salud. Sin embargo, este estudio nos da un punto de partida para abordar las desigualdades en materia de salud, que siguen siendo urgentes tanto en Argentina como en Haiti. Medir el problema, establecer metas para progresar y crear mecanismos de rendición de cuentas son herramientas ampliamente reconocidas de una planificación eficaz; y los datos muestran que estas herramientas no están debidamente utilizadas para abordar la equidad en la salud.

Este análisis se enfrentó a varias limitaciones. En primer lugar, existe una limitación inherente al tratar de comprender el entorno de política y las acciones de un país para promover la equidad en la salud mediante el examen de planes documentados, porque existen otras leyes y políticas que afectan la equidad en la salud y porque los planes de salud y de desarrollo de los países solo pueden entenderse plenamente en sus contextos políticos, institucionales y sociales generales, incluidos los progresos ya realizados hacia una mayor equidad en la salud. Por lo tanto, los modestos objetivos de este estudio no deben interpretarse como absolutos. El análisis futuro de un conjunto más amplio de documentos jurídicos y de políticas podría resultar fructífero para tener un panorama más amplio. Además, los países adoptan diferentes enfoques sobre el nivel de especificidad y detalle, lo que refleja que estos hallazgos están influenciados por las características más amplias del proceso de planificación y la documentación en un país determinado. Dicho esto, la teoría en la que se basa este trabajo es que la medición, la planificación y la rendición de cuentas pueden ser importantes para mejorar la equidad en la salud, y estos resultados aportan una representación inicial de la atención nacional que se presta a esos factores.

## Conclusiones

El compromiso casi unánime de los países de la Región de las Américas con la equidad en la salud, expresado en sus planes nacionales de salud, ofrece una importante oportunidad para avanzar hacia la eliminación de la inequidad. Sin embargo, hemos encontrado grandes variaciones en cuanto a la sustancia y la estructura con que se aborda este tema en que los planes de salud en la Región.

Resulta útil que, en muchos países, se disponga de datos de referencia en los planes nacionales sobre varios ejes de desigualdad, contra los cuales se podría juzgar el avance. En otros países, estos datos de referencia serán necesarios para una planificación real que ayude a propiciar la equidad. La voluntad política de traducir los objetivos en efectos se verá en los próximos años si es que se establecen y alcanzan objetivos con plazos específicos. Hasta ahora, menos de la mitad de los países han incluido metas con plazos específicos sobre la reducción de las desigualdades absolutas o relativas n la salud, lo que probablemente socavará los progresos en materia de equidad.

Algunas políticas más sustantivas enfocadas en la salud, como las que mejoran la accesibilidad física de la atención de salud y aumentan el acceso asequible a los medicamentos, se incluyen en muchos planes, aunque ningún país incluye todos los aspectos examinados. Los procesos participativos documentados en la formulación de estos planes van de inexistentes hasta sólidos y amplios con respecto a las políticas, fijación de objetivos y rendición de cuentas incluidos en sus planes de salud. Esto sugiere que tanto el apoyo técnico al logro de la equidad en la salud al elaborar los planes, como el aprendizaje entre pares, podrían ser beneficiosos para respaldar la planificación a fin de alcanzar los objetivos declarados. Además, subsisten brechas en cuanto a las acciones para abordar las inequidades en los grupos poblacionales marginados, en especial la población afrodescendiente, los pueblos indígenas y los migrantes.

La diversidad de planes presenta una excelente oportunidad de aprendizaje. Dado que algunos países han creado planes detallados y bien concebidos para lograr la equidad, se podrían aprovechar varias ideas. Pero no hay dos planes iguales, e incluso los países con planes más sólidos podrían inspirarse en otros.

El marco que se elaboró para este estudio representa un paso hacia la evaluación y comprensión del entorno de políticas para la equidad en la salud que podría aplicarse, en trabajos futuros, a una gama más amplia de políticas, leyes y estrategias de salud. Esto también puede ser útil en trabajos futuros para entender qué tipo de políticas son particularmente eficaces y apoyan el aprendizaje regional. La formulación de políticas es una intervención —destinada a llevar las ideas a una escala nacional—, y someterla a revisión, evaluación y mejora puede ayudar a cumplir la ambición generalizada de reducir las desigualdades en toda la Región.

## Declaración.

Los autores son los únicos responsables de las opiniones expresadas en el manuscrito, que no necesariamente reflejan la opinión o la política de la RPSP/PAJPH o de la OPS.

## Footnotes

1. En el 2017, Grogan realizó un análisis de la equidad en la salud en la ley de atención de salud asequible de Estados Unidos (ACA, por su sigla en inglés) (23). Sin embargo, dado que la ACA es una legislación sanitaria con implementación descentralizada, en lugar de un plan nacional de salud, y está principalmente (aunque no de manera exclusiva) centrada en el financiamiento de la atención médica, en especial el seguro de salud, no es adecuado incluirla en esta comparación.
